# Insects in agricultural greenhouses: a metagenomic analysis of microbes in *Trialeurodes vaporariorum* infesting tomato and cucumber crops

**DOI:** 10.3389/fpls.2025.1581707

**Published:** 2025-05-19

**Authors:** Abeer Qush, Nada Assaad, Fatima Abdulla Alkhayat, Mohammed Saif Al-Kuwari, Nasser Al-Khalaf, Maya Bassil, Hadi M. Yassine, Asad Zeidan, Rozaimi Razali, Layla Kamareddine

**Affiliations:** ^1^ Department of Biomedical Sciences, College of Health Sciences, QU Health, Qatar University, Doha, Qatar; ^2^ Department of Biological and Environmental Sciences, College of Arts and Sciences, Qatar University, Doha, Qatar; ^3^ Ministry of Environment and Climate Change, Doha, Qatar; ^4^ Agrico for Agricultural Development Company, Doha, Qatar; ^5^ Department of Nutrition Sciences, College of Health Sciences, QU Health, Qatar University, Doha, Qatar; ^6^ Biomedical Research Center, QU Health, Qatar University, Doha, Qatar; ^7^ Department of Basic Medical Sciences, College of Medicine, QU Health, Qatar University, Doha, Qatar; ^8^ The KINDI Center for Computing Research, College of Engineering, Qatar University, Doha, Qatar

**Keywords:** greenhouse systems, insects, *Trialeurodes vaporariorum*, meteorological conditions, metagenomics, agricultural yield

## Abstract

**Introduction:**

With the predicted 9-10 billion world population increase by 2050 and its accompanying need for sustainable food production, and with the harsh climate conditions challenging agriculture and food security in many countries world-wide, employing “horticultural protected cultivation practices” in farming for seasonal and off-seasonal crop production is on the rise, among which is the use of agricultural greenhouses. The importance of greenhouse farming has been, indeed, evident by the perceived increase in year-round crops production, curtail in production risks, upsurge in agricultural profits, outreaching food stability and security in many countries globally. Yet, and despite this acknowledged success of employing greenhouses in farming, many constraints, including the presence of insect pests, still chaperoned this practice over the years, significantly impacting crop quality and production.

**Methods:**

As such, we assessed in this study the status of “insect pests” in the greenhouse model by collecting insects from different greenhouse sectors grown with tomatoes and cucumbers and identified the collected insects using relevant identification keys. To further explore the pest paradigm in greenhouses, we then focused on particularly studying *Trialeurodes vaporariorum* (*TRIAVA*), a key insect species among the collected and identified insects in the studied greenhouse model and a significant pest with an impactful effect on many crops worldwide. To do so, we traced the abundance of *TRIAVA* in the tomato and cucumber grown greenhouse sectors over the period of the study, analyzed its metagenome and associated its abundance with crop yield.

**Results and discussion:**

Our findings revealed *TRIAVA* hosted microbes with aptitudes to either serve as symbiotic microorganisms and protect *TRIAVA* against pathogens or to potentially cause damage to crops. This work provides additional insight into the insect pests paradigm in greenhouses, an upshot that could serve integrated insect pest management strategies in greenhouses for optimal agricultural practices.

## Highlights

- Landscape of “insect pests” in agricultural greenhouse systems.
- *Trialeurodes vaporariorum* abundance in agricultural greenhouses.
- *Trialeurodes vaporariorum* metagenomic analysis.
- *Trialeurodes vaporariorum* impact on crop yield.

## Introduction

For the past decades, and despite the substantial progress made in combating global hunger, malnutrition and food insecurity persisted as far-reaching problems in many countries worldwide, mainly in Asia and Africa (Foley et al., 2011; Global Panel on Agriculture and Food Systems for Nutrition, 2016; International Food Policy Research Institute (IFPRI), 2016; FAO, I et al., 2017). While poverty classifies as a major motive, the ground for food insecurity outstretches far beyond poverty to encompass other additional motives including the rapidly growing human population worldwide, climate change, and weather conditions. These motives also exhibit serious threats to farming practices and agricultural sustainability (Farooq et al., 2019). Being a major food security pillar, the necessity to develop multidimensional farming approaches for agricultural sustainability without impacting economy, society, and environmental integrity becomes therefore imperative. Among various technological innovations, the use of “controlled-environment-agricultural-production-technologies” such as greenhouse farming has been promising in overcoming production related obstacles, increasing productivity rates and securing agricultural sustainability (Benke and Tomkins, 2017; Forkuor et al., 2022). Compared to open field farming practices, greenhouse cultivation has been shown to upsurge crop yields, decrease the release and accumulation of greenhouse gases, promote an efficient use of agricultural resources such as water, nutrients and energy, and curtail the stressing effect of some abiotic and biotic factors (Kanwar, 2011). Despite these advantages; however, several challenges still acquainted the use of this innovative technology. Among those challenges, pest manifestation has been considered a paramount factor in causing drastic losses in crop yield, if left uncontrolled. Agricultural pests span a broad-spectrum including insects/insect vectors, weeds, rodents, nematodes, mites, microorganism and viruses (Nriagu, 2011; Heck, 2018; Walker and Frederick, 2019; Vermelho et al., 2024). Indeed, insect vectors have been reported to cause one of the greatest threats not only to crops but also to animals and humans on a global scale (Kamareddine, 2012; Heck, 2018). Most insect vectors carrying plant pathogens classify as hemipteran insects, examples of which include psyllids, aphids and whiteflies (Heck, 2018). Interestingly, many of these insect vectors have been identified in agricultural greenhouses. The *Trialeurodes vaporariorum* (*TRIAVA*) white fly pest, for example, has been reported to pose serious threats to roses cultivated in greenhouses (Roditakis, 1990; Voigt et al., 2019) and to transmit pathogens like the *Tomato torrado virus* (Verbeek et al., 2014) and the *Pepino mosaic virus* (Noël et al., 2014) to tomato crops causing severe necrosis and crop loss (van der Vlugt et al., 2000; Wieczorek et al., 2020). Likewise, aphids have been shown to significantly impact different crops including cucumber, eggplant, tomato and potato grown in greenhouses (Eastop and Balckmann, 2007; Capinera, 2008; Tarek, 2013; Mohammed et al., 2018; Mohammed et al., 2022). Aphids have been, indeed, elegantly outlined for their roles as vector. Among various aphid-transmitted plant virus families, the family *Potyviridae*, genus *Potyvirus*, is probably considered the most important based on the exceptionally large number of virus species that it includes (Qin et al., 2020). Furthermore, the *Bactericera cockerelli* psyllid, known to transmit *Candidatus* Liberibacter solanacearum, has been also reported as solanaceous crops (tomato and potato) insect pest (Rosson et al., 2006; Yang and Liu, 2009; Munyaneza, 2012; Munyaneza, 2013; Castillo Carrillo, 2019; Tang et al., 2020; Sarkar et al., 2023). It is noteworthy here that despite classifying as vectors, many of those insect pests do not necessarily exclusively cause crop damage by means of the pathogen they carry, but could rather exhibit pathogenicity via other mechanisms or by the combination of multiple-damaging strategies. The impact of whiteflies, for instance, on crop damage or yield loss is not only attributed to the pathogens carried by the insect, but also to the insect’s direct feeding effect on the plant, along with the potentiality of this feeding behavior to attract other pathogens (Navas-Castillo et al., 2011; Saurabh et al., 2021).

**Figure 1:**
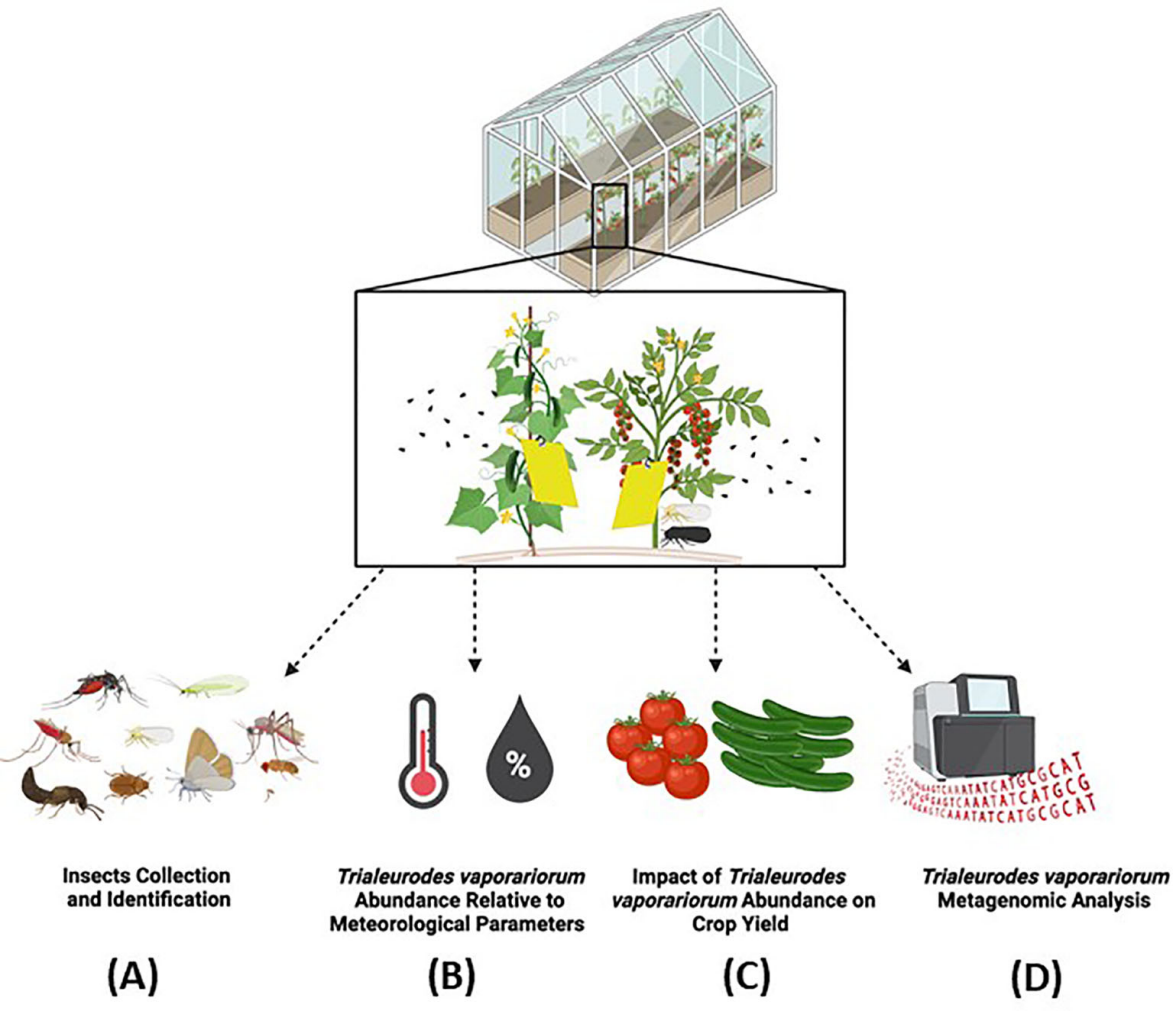
Study overview. In this study **(A)** agricultural greenhouse insects were collected and identified, *Trialeurodes vaporariorum* abundance was scored for and associated with **(B)** meteorological parameters and **(C)** cucumber and tomato crop yield, and **(D)** *Trialeurodes vaporariorum* metagenome was analyzed. Biorender was used to generate the study overview diagram.

Owing to the effect of insect pests on greenhouse farming and to the existence of such multiple-plant/crop damaging mechanisms, a proper and comprehensive understanding of the landscape of insect pests in agricultural greenhouses and their virulence mechanisms is crucial for the development of either in isolation or in combination strategies for insect pests control. As such, we conducted in this study an “insects survey” to detect the present and most abundant insect pests in selected agricultural greenhouse sectors grown with tomato and cucumber. Being among the mostly detected key insect species in our studied greenhouse model and a major global pest, we further scored for the abundance of *TRIAVA*, in particular, and evaluated the association of its abundance with the greenhouse environmental conditions including temperature and humidity. We also conducted a metagenomics analysis to detect the richness and diversity of the microbial communities residing in *TRIAVA* and to assess the potential contribution of those microbes to vector protection and crop yield loss using computational predictions (**Figure 1**). Taken together, our findings provide novel insights into the insect pests paradigm in agricultural greenhouses, and its associated biotic and abiotic framework. By focusing on such paradigms, the agricultural community can work towards a more efficient, sustainable, and cost-effective future, securing a steady supply of crops while safeguarding the ecosystem.

## Materials and methods

### Agricultural greenhouses

The insects survey was conducted in three greenhouses located in the AGRICO Agricultural Development W.L.L. farm that is situated in Al-Khor city, 58 km away from Doha the capital of Qatar, and that covers an estimated area of 24 hectares (https://agrico.qa/). Cucumbers were grown in an arch-shaped, one side, UV coated greenhouse made of an 8mm clear polycarbonate hollow sheet. Cherry and Beef tomatoes were grown in arch-shaped greenhouses covered with 200 microns-white plastic sheets, with a 7% UV light penetration ability. For close monitoring of the environmental conditions inside the agricultural greenhouses, meteorological data including temperature (°C), relative humidity (%), vapor pressure deficit (kPa), evapotranspiration (mm d^-1^), solar radiation/photosynthetically active radiation (Wm^-2^), and wind velocity (km/h) were recorded at fixed intervals using the Crop Estimation through Resource and Environment Synthesis monitoring system (CERES, USA).

### Insects collection and identification

The insects survey was conducted during the planting season, particularly during the period extending from the 19^th^ of February to the 11^th^ of June 2022. Over the indicated period, insects were collected on weekly basis from selected 3000-4000m^2^ agricultural greenhouse sectors grown with tomato or cucumber. The collection methods included UV light traps (BIONIVA, Malaysia), mechanical pooters/aspirators (Labitems, India), yellow sticky traps, and handpicking. Collected insects were placed in Eppendorf or Falcon conical tubes, transported on ice from the farm to the laboratory, and stored at -80°C for identification. Using available literature and taxonomic keys (Diarra et al; Kelton, 1975; Rattanarithikul, 1982; Abdu and Shaumar, 1985; Harbach, 1985; Aldryhim, 1996; Martin et al., 2000; Beadle and Leckie, 2012; Gibb and Oseto, 2019; Garba et al., 2020; Dioli et al., 2021; Lonsdale, 2021), and under a stereo microscope (Leica M125), insects were identified up to the species level whenever possible and representative images were taken.

### *Trialeurodes vaporariorum* abundance monitoring

To monitor *TRIAVA* density throughout the planting season in the studied sectors, 15cm x 20cm (width x length) yellow sticky traps were distributed into five selected spots in each tomato and cucumber grown sector. To obtain unbiased mean density and ensure that sticky traps are spatially independent, traps were placed at 18m apart in 3000m^2^ sector and at 22.5m apart in 4000m^2^ sector, as recommended from previous studies (Kim et al., 2001). On weekly basis, old traps were replaced by new ones and *TRIAVA* counts were recorded.

### Data analysis

Descriptive statistics were used to present the meteorological data, particularly temperature (°C) and relative humidity (%), recorded in the tomato and cucumber grown greenhouse sectors across the period of the study. Pearson’s correlation was used to associate *TRIAVA* abundance with meteorological data and crop yield. The IBM SPSS Statistics software V22 was used to conduct data analysis between compared groups and statistical significance was concluded for p-value ≤ 0.05.

### Metagenomic analysis

The metagenome analysis workflow involved multiple steps including sample preparation, quality control and functional annotation, the details of each step are summarized as follows:

#### Sample preparation and DNA library construction and sequencing

During the study period, extending from the 19^th^ of February to the 11^th^ of June 2022, 3-5 *TRIAVA* insects per sample were collected at different times from different spots of the studied sectors using a mechanical aspirator. Samples were then pooled to make up a total of 16 insects per pooled sample and surface sterilized before DNA extraction. *TRIAVA* genomic DNA was then extracted using phenol-chloroform at Beijing Genomics Institute (BGI) (BGI Genomics, 2021). In brief, tissues samples were grinded with liquid nitrogen, suspended in 1 mL of lysis buffer, incubated at 56°C for 30-180 minutes with continuous mixing by inversion every 5-10 minutes, and centrifuged at 16700×g for 10 minutes. The supernatant was then transferred to a new tube and mixed with an equal volume of chloroform/isoamyl alcohol (24:1). The aqueous phase was then collected and transferred into a new tube, mixed with an equal volume of isopropyl alcohol, and centrifuged at 16700×g for 10 minutes. The aqueous phase was further collected and transferred into a new tube, mixed with 2/3 volume of isopropyl alcohol, incubated at -20°C for 2 hours for precipitation, and centrifuged at 16700×g for 10 minutes. The DNA pellet was then washed twice with 75% ethanol, centrifuged, air-dried, and resuspended in 25-100 µL of TE buffer. The quality and quantity of the extracted samples was then assessed using a Qubit fluorometer and agarose gel electrophoresis. To prepare the DNA libraries, 1μg of genomic DNA was randomly fragmented, and the fragmented DNA was size-selected using the Agencourt AMPure XP kit to an average of 200–400 bp. Fragments were end repaired and 3’ adenylated. Adaptors were further ligated to the DNA fragments then amplified. The PCR products were purified using the Agencourt AMPure XP kit. The double-stranded PCR products were heat-denatured and circularized using a splint oligonucleotide. The single-stranded circle DNA (ssCir DNA) was formatted as the final library. The Library was validated through quality control. The qualified libraries were then sequenced by BGISEQ-500_ DNBSEQ™ and through rolling-cycle replication, ssCir DNA molecule formed a DNA nanoball library (DNB). DNBs were next loaded onto the patterned nanoarray by using high-density DNA nanochip technology and pair-end 100 bp reads were finally obtained through combinatorial Probe-Anchor Synthesis (cPAS).

#### Quality control and preprocessing

To evaluate the overall quality, including base quality scores, sequence length distribution, and potential contamination, quality control of raw sequencing reads was performed using FastQC (Andrews et al., 2010). To expedite the process, the analysis was conducted using 30 threads, and the results were saved in the output directory. Following this, Fastp (Chen, 2023) was used to trim low-quality bases, remove adapter sequences, and improve read quality. The paired-end input files were processed with 30 threads, and the cleaned reads were output to the output directory for downstream analysis.

#### Removal of human reads

To ensure that only non-human reads were retained, the trimmed reads were mapped to the human reference genome (GRCh38) using Bowtie2 (Langmead and Salzberg, 2012). To maximize read alignment precision, mapping was performed with a very-sensitive alignment setting. Reads that aligned to the human genome were filtered out, while non-human reads were extracted and saved for subsequent taxonomic classification. The unmapped reads were considered for further downstream analysis.

#### Taxonomic classification

To determine the taxonomic composition of the metagenome, Kraken2 (Lu et al., 2022), a database-driven classifier that assigns taxonomic labels based on exact k-mer matches, was used. The analysis was performed with a minimum base quality threshold of 30 and a requirement for at least two hit groups to reduce false-positive classifications. The classified and unclassified reads were saved in respective files for later inspection. A detailed report (trv.k2report) was also generated to provide an overview of the classified taxa.

#### Abundance estimation and filtering

To estimate taxonomic abundances from Kraken2 output at various levels including kingdom (K), domain (D), phylum (P), class (C), order (O), family (F), genus (G), and species (S), we used Bracken (Lu and Salzberg, 2019). Using a custom Python script, filtering to remove specific taxa that were considered contaminants or non-target organisms was performed. The filtered output files were saved with relevant taxonomic breakdowns, ensuring that only taxa of interest were retained for analysis.

#### Taxonomic visualization

To visualize the taxonomic distribution of the metagenome, Krona (Ondov et al., 2011) plots were generated. The kreport2krona.py script converted the Bracken report into a Krona-compatible format, and the ktImportText tool was used to generate an interactive HTML visualization, providing an intuitive representation of the relative abundance of each taxonomic group.

#### Gene prediction

To conduct gene prediction, MetaGeneMark (Gemayel et al., 2022), a robust tool for identifying coding sequences (CDS) in metagenomic assemblies, was used. The analysis utilized the MetaGeneMark v1 model, and both nucleotide and protein-coding sequences were extracted. The predicted proteins and nucleotide sequences were output for subsequent functional annotation and downstream comparative analysis.

#### Functional annotation

The predicted nucleotide sequences were annotated using BLASTn (Geneious) by querying the NCBI nucleotide database. The search was performed with an e-value cutoff of 1×10^−51^ times to ensure significant alignments, and the output was formatted in tabular format (format 6) to facilitate interpretation. Only the top match for each query sequence was retained to avoid redundancy, and the analysis was parallelized using 30 threads to improve computational efficiency.

#### Gene Ontology mapping

For functional characterization, Gene Ontology (GO) terms associated with the annotated genes were retrieved. A custom Python script was employed to map GO terms by querying the QuickGO API (European Bioinformatics Institute) and fetching descriptions for each GO term. The input file containing GO terms was processed, and the output was saved as a tab-separated values (TSV) file. A brief delay was included between API requests to avoid exceeding the rate limit.

## Results

### Studied greenhouse sectors and recorded meteorological datasets

**Figure 2:**
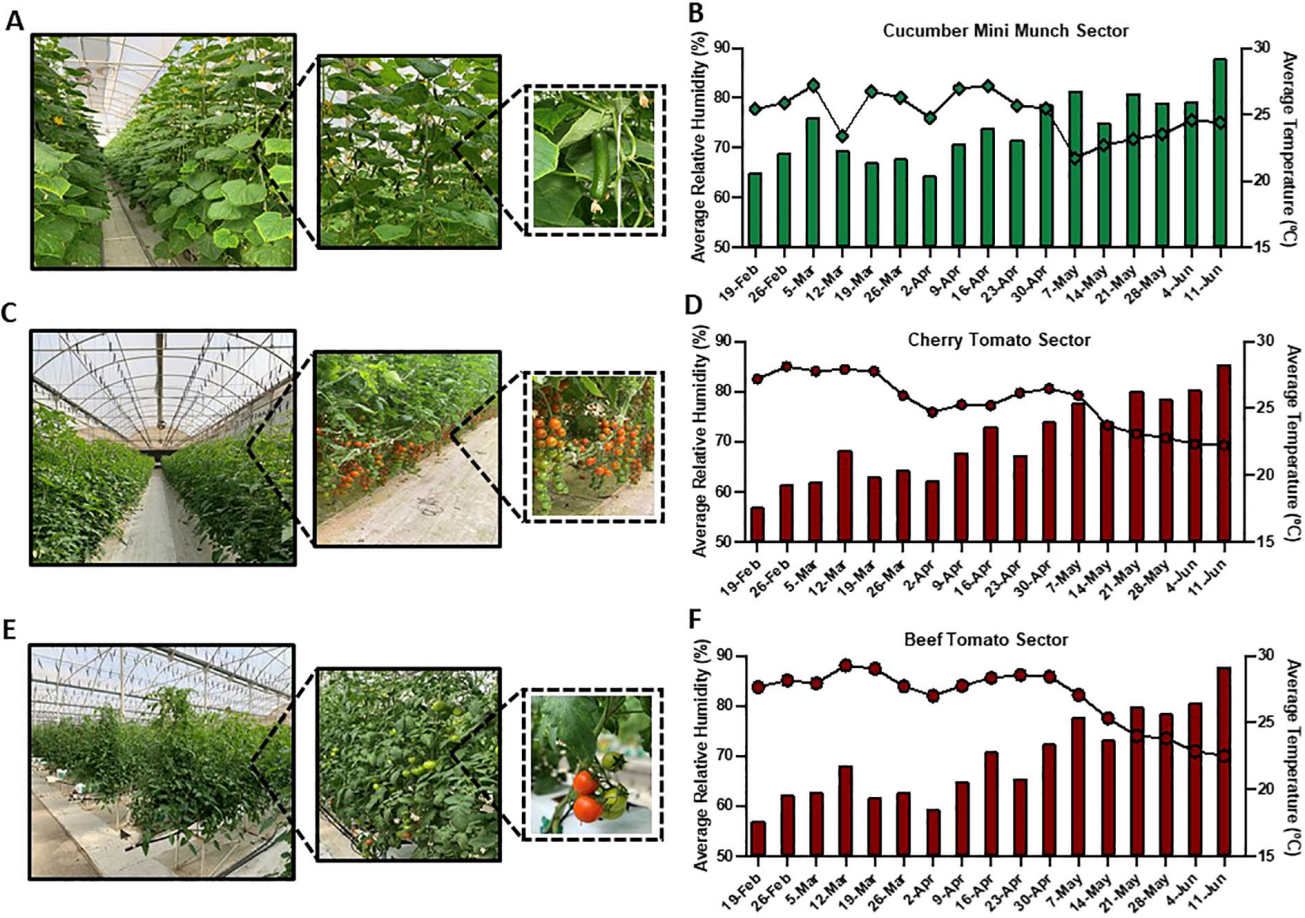
Greenhouse crops and meteorological conditions. Representative images of the studied agricultural greenhouse sectors grown with **(A)** Cucumber Mini Munch **(C)** Cherry Tomato and **(E)** Beef Tomato. Bar graphs representing the weekly average temperature and line charts demonstrating the weekly average relative humidity in **(B)** Cucumber Mini Munch **(D)** Cherry Tomato and **(F)** Beef Tomatc grown sectors recorded during the period of the study.

Although ambient conditions are relatively maintained in agricultural greenhouses as compared to open fields, variations in meteorological conditions, particularly in temperature and relative humidity, could still occur due to natural and static driven factors (van Tuijl et al., 2008; Balendonck et al., 2010). In this study, we chose to particularly focus on greenhouse sectors grown with cucumber and tomato, including Cucumber Mini Munch (**Figure 2A**), Cherry Tomato (**Figure 2C**), and Beef Tomato (**Figure 2E**), being essential horticultural crops grown in greenhouses globally (Xue et al., 2023). We also recorded the meteorological datasets, specifically temperature (°C) and relative humidity (%), in those sectors throughout the period of the study. In concordance with the reported from previous findings, slight variations in temperature and relative humidity readouts throughout the period of the study have been, indeed, detected in these monitored greenhouse sectors (**Figures 2B, D, F**). Along this, slight variations in the temperature and relative humidity readouts between the three studied sectors were also recorded. For example, in the Cucumber Mini Munch sector, the average temperature levels fluctuated from a minimum of 20.4°C to a maximum of 29.2°C, and the average relative humidity levels from a minimum of 67.9% to a maximum of 82.5% (**Figure 2B**), reflecting a slightly higher minimum average temperature and a slightly lower minimum average humidity levels compared to those recorded in the two other sectors. In the Cherry Tomato sector, on the other hand, average temperature ranged from a minimum reading of 17.6°C to a maximum reading of 28.3°C, while average relative humidity varied from a minimum of 69.3% to a maximum 85% (**Figure 2D**). Similarly, the recorded average temperature values in the Beef Tomato sector ranged from a minimum reading of 17.6°C to a maximum reading of 29.2°C, and the average relative humidity levels fluctuated from a minimum of 70% to a maximum of 88.1% (**Figure 2F**).

### Collected and identified insects

**Figure 3:**
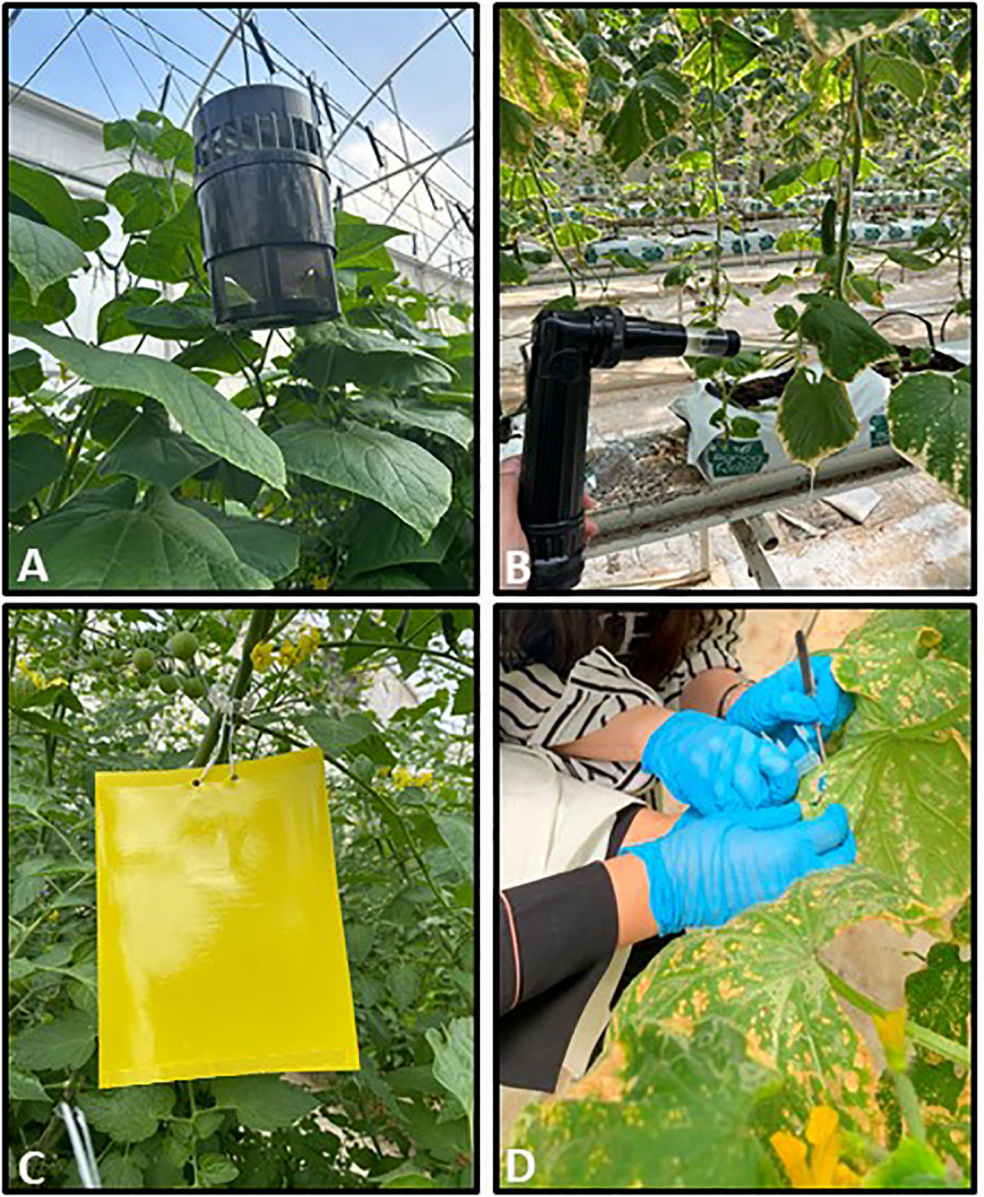
Insects collection tools. Images of the tools including **(A)** UV light trap, **(B)** mechanical pooter (aspirator), **(C)** yellow sticky trap, and **(D)** handpicking used for insects collection from Cucumber Mini Munch, Cherry Tomato, and Beef Tomato grown greenhouse sectors.

Monitoring and controlling the dynamics of pest population in greenhouses is paramount in efficiently managing greenhouse farming. Herein, we performed greenhouse insects (beneficial or pests) investigation by collecting insects residing in the studied cucumber and tomato grown greenhouse sectors using readily available tools (**Figure 3**). Collected insects were further identified up to the species level expect for a few cases where identification was only achieved up to the genus level (**Table 1**; **Figure 4**). Failure to complete species-level classification was either due to the lack of local and regional taxonomic keys or to damages that have occurred to some samples during the collection, transferring and/or characterization processes. Our findings revealed a total of 42 insects belonging to different orders, including Diptera, Coleoptera, Hemiptera, Lepidoptera, and Neuroptera (**Table 1**; **Figure 4**). Among those insects, *Liriomyza trifolii*, *Trialeurodes vaporariorum*, *Dicranomyia modesta*, *Nomophila noctuella*, *Spodoptera exigua*, and *Ceraeochrysa* were the most prevalent during the study period. Interestingly, a remarkable increase in the insects’ overall abundance in general was also detected from May to June.

**Table 1:** List of collected insects from Cucumber Mini Munch, Cherry Tomato, and Beef Tomato grown greenhouse sectors during the period of the study.

#	Order	Family	Genus	Species	Collection Method
1	Diptera	Agromyzidae	*Liriomyza*	*Liriomyza trifolii*	Light Trap, Handpicking
2	Diptera	Chironomidae	*Chironomus*	*_*	Light Trap
3	Diptera	Chironomidae	*Ablabesmyia*	*_*	Light Trap
4	Diptera	Culicidae	*Aedes*	*Aedes caspius*	Light Trap
5	Diptera	Culicidae	*Anopheles*	*Anopheles stephensi*	Light Trap
6	Diptera	Culicidae	*Culex*	*Culex perexiguus*	Light Trap
7	Diptera	Culicidae	*Culex*	*Culex quinquefasciatus*	Light Trap
8	Diptera	Dolichopodidae	*Chrysotus*	*Chrysotus neglectus*	Light Trap
9	Diptera	Drosophilidae	*Drosophila*	*Drosophila melanogaster*	Light Trap
10	Diptera	Drosophilidae	*Zaprionus*	*Zaprionus indianus*	Light Trap
11	Diptera	Psychodidae	*Psychoda*	*_*	Light Trap
12	Diptera	Sciaridae	*Bradysia*	*Bradysia fungicola*	Light Trap
13	Diptera	Limoniidae	*Dicranomyia*	*Dicranomyia modesta*	Light Trap
14	Coleoptera	Cryptophagidae	*Cryptophagus*	*Cryptophagus dentatus*	Light Trap
15	Coleoptera	Curculionidae	*Hylastes*	*Hylastes cunicularius*	Light Trap
16	Coleoptera	Mycetophagidae	*Typhaea*	*Typhaea stercorea*	Light Trap
17	Coleoptera	Scarabaeidae	*Aphodius*	*_*	Light Trap
18	Coleoptera	Staphylinidae	*Stamnoderus*	*_*	Light Trap
19	Coleoptera	Staphylinidae	*Hydrosmecta*	*_*	Light Trap
20	Hemiptera	Aleyrodidae	*Trialeurodes*	*Trialeurodes vaporariorum*	Handpicking, Aspirator, Yellow Sticky Trap
21	Hemiptera	Aphididae	*Aphis*	*Aphis gossypii*	Handpicking
22	Hemiptera	Lygaeidae	*Remaudiereana*	*Remaudiereana annulipes*	Light Trap
23	Hemiptera	Miridae	*Creontiades*	*Creontiades pallidus*	Light Trap
24	Hemiptera	Miridae	*Lygus*	*Lygus elisus*	Light Trap
25	Hemiptera	Miridae	*Macrolophus*	*Macrolophus caliginosus*	Light Trap
26	Hemiptera	Miridae	*Nesidiocoris*	*Nesidiocoris tenuis*	Light Trap, Handpicking
27	Hemiptera	Reduviidae	*Empicoris*	*Empicoris rubromaculatus*	Light Trap
28	Lepidoptera	Crambidae	*Diaphania*	*Diaphania indica*	Light Trap
29	Lepidoptera	Crambidae	*Nomophila*	*Nomophila noctuella*	Light Trap
30	Lepidoptera	Crambidae	*Spoladea*	*Spoladea recurvalis*	Light Trap
31	Lepidoptera	Erebidae	*Utetheisa*	*Utetheisa pulchella*	Light Trap
32	Lepidoptera	Geometridae	*Scopula*	*Scopula* *ochroleucaria*	Light Trap
33	Lepidoptera	Noctuidae	*Mythimna*	*Mythimna unipuncta*	Light Trap
34	Lepidoptera	Noctuidae	*Spodoptera*	*Spodoptera* *littoralis*	Light Trap
35	Lepidoptera	Noctuidae	*Spodoptera*	*Spodoptera* *exigua*	Light Trap
36	Lepidoptera	Noctuidae	*Trichoplusia*	*_*	Light Trap
37	Lepidoptera	Nolidae	*Earias*	*Earias insulana*	Light Trap
38	Lepidoptera	Nolidae	*Meganola*	*Meganola albula*	Light Trap
39	Lepidoptera	Oecophoridae	*Aeolothapsa*	*Aeolothapsa malacella*	Light Trap
40	Lepidoptera	Pyralidae	*Pyralis*	*Pyralis pictalis*	Light Trap
41	Lepidoptera	Gelechiidae	*Scrobipalpa*	*Scrobipalpa* *ocellatella*	Light Trap
42	Neuroptera	Chrysopidae	*Ceraeochrysa*	*_*	Light Trap

**Figure 4:**
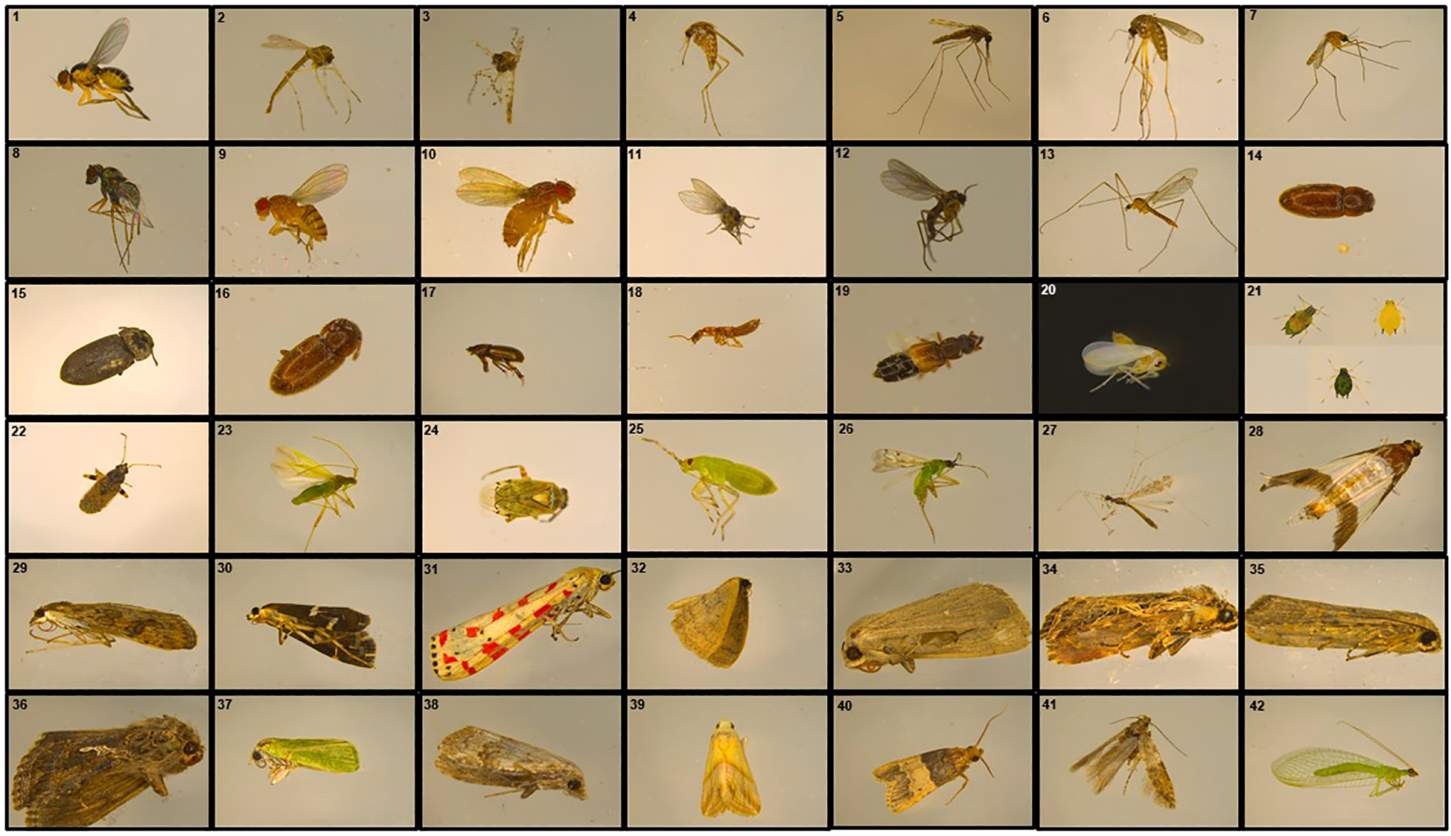
Collected insects. Representative images of (1) *Liriomyza trifolii*, (2) *Chironomus*, (3) *Ablabesmyia*, (4) *Aedes caspius*, (5) *Anopheles stephensi*, (6) *Culex perexiguus*, (7) *Culex quinquefasciatus*, (8) *Chrysotus neglectus*, (9) *Drosophile melanogaster*, (10) *Zaprionus indianus*, (11) *Psychoda*, (12) *Bradysia fungicola*, (13) *Dicranomyia modesta*, (14) *Cryptophagus dentatus*, (15) *Hylastes cunicularius*, (16) *Typhaea stercorea*, (17) *Aphodius*, (18) *Stamnoderus*, (19) *Hydrosmecta*, (20) *Trialeurodes vaporariorum*, (21) *Aphis gossypii*, (22) *Remaudiereana annulipes*, (23) *Creontiades pallidus*, (24) *Lygus elisus*, (25) *Macrolophus caliginosus*, (26) *Nesidiocoris tenuis*, (27) *Empicoris rubromaculatus*, (28) *Diaphania indica*, (29) *Nomophila noctuella*. (30) *Spoladea recurvalis*, (31) *Utetheisa pulchella*, (32) *Scopula ochroleucaria*, (33) *Mythimna unipuncta*, (34) *Spodoptera littoralis*, (35) *Spodoptera exigua*, (36) *Trichoplusia*, (37) *Earias insulana*, (38) *Meganola albula*, (39) *Aeolothapsa malacella*, (40) *Pyralis Pyralis pictalis*, (41) *Scrobipalpa ocellatella*, and (42) *Ceraeochrysa* collected from Cucumber Mini Munch, Cherry Tomato, and Beef Tomato grown greenhouse sectors during the period of the study.

### *Trialeurodes vaporariorum* abundance and impact on crop yield

**Figure 5:**
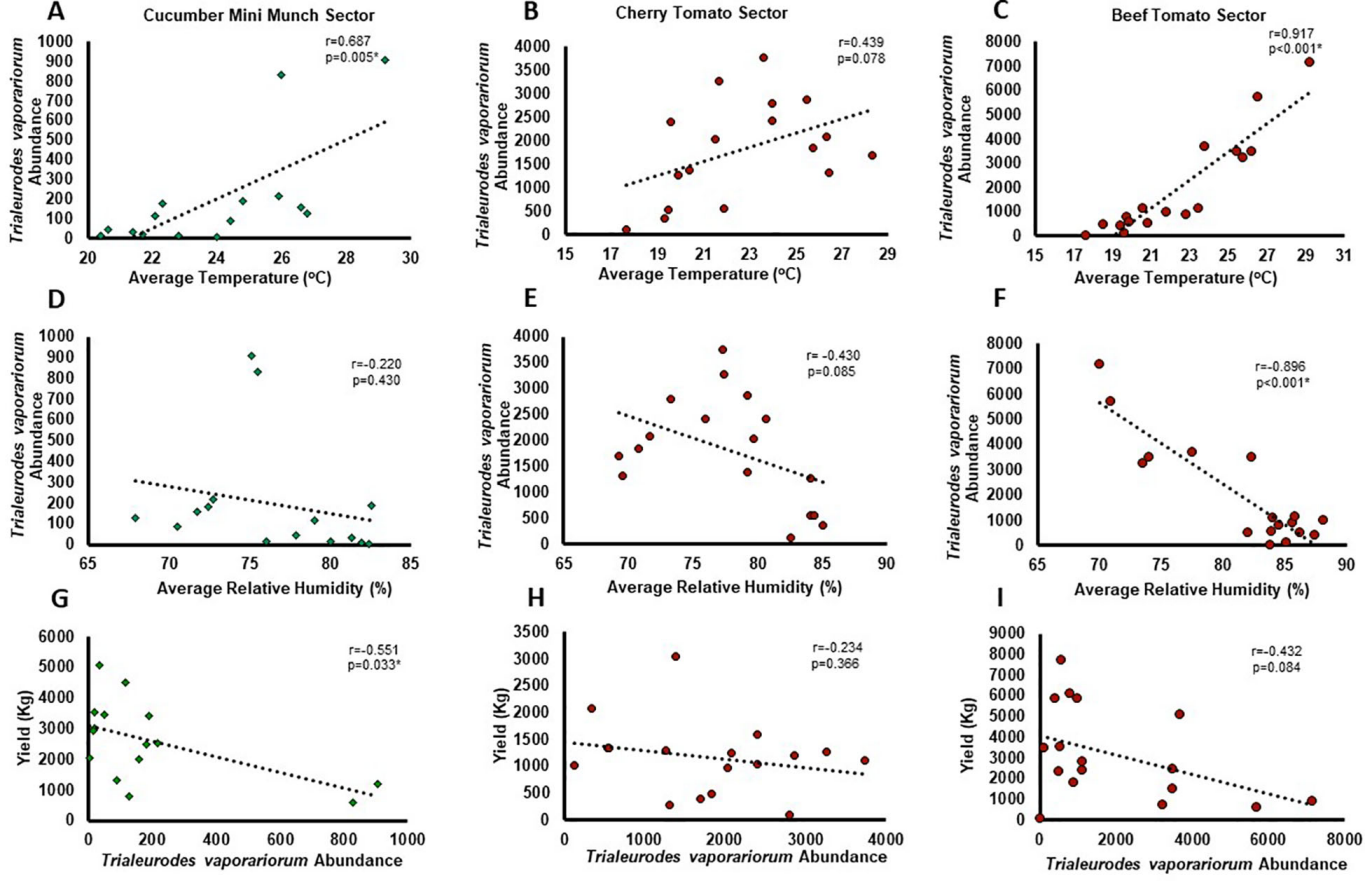
Correlation between *Trialeurodes vaporariorum* abundance and greenhouse meteorological conditions and crop yield. Scatter diagram and best-fit linear model representing the correlation between average *Trialeurodes vaporariorum* weekly abundance and **(A-C)** average weekly temperature, **(D-F)** average weekly relative humidity, and **(G-I)** average weekly crop yield recorded throughout the period of the study in **(A, D, G)** Cucumber Mini Munch, **(B, E, H)** Cherry Tomato, and **(C, F, I)** Beef Tomato grown sectors. Pearson correlation coefficient (r) and p-value are shown on each graph, with positive r-value signifying positive correlation, negative r-value signifying negative comelation, and p-value ≤ 0.05 indicating statistical significance.

Among the identified insects in the studied greenhouse sectors (**Table 1**, **Figure 4**), *TRIAVA* was one of the most abundant pest species known to affect vegetables and ornamental greenhouse crops and cause economical losses (Wraight et al., 2017). As such, we next evaluated the abundance of the *TRIAVA* whitefly in these sectors throughout the period of the study and correlated its abundance with meteorological parameters, particularly with temperature and relative humidity, being pivotal factors in dictating pest plentitude and distribution (Kamareddine, 2019; Khan, 2019). Subsequently, we further correlated *TRIAVA* abundance with cucumber and tomato crop yield. Our findings revealed an overall positive correlation between *TRIAVA* abundance and temperature (**Figures 5A–C**) and negative correlation between *TRIAVA* abundance and relative humidity in the three studied sectors (**Figures 5D–F**). Moreover, our findings also revealed that the total weekly crop yield fluctuates in an inversely proportional manner with *TRIAVA* abundance, as evidenced by the negative correlation between *TRIAVA* abundance and crop yield in the three studied sectors (**Figures 5G–I**). It is worth noting here that despite the clear correlation trends seen between *TRIAVA* abundance and the studied meteorological conditions and crop yield, boarded line or ≥ 0.05 p-values have been concluded in some occurrences. Failure to achieve statistical significance in these occurrences does not negate the existing trend and could rather relate to many reasons, one of which might be the low whitefly counts sometimes recorded in these studied sectors.

### *Trialeurodes vaporariorum* metagenomic analysis

**Figure 6:**
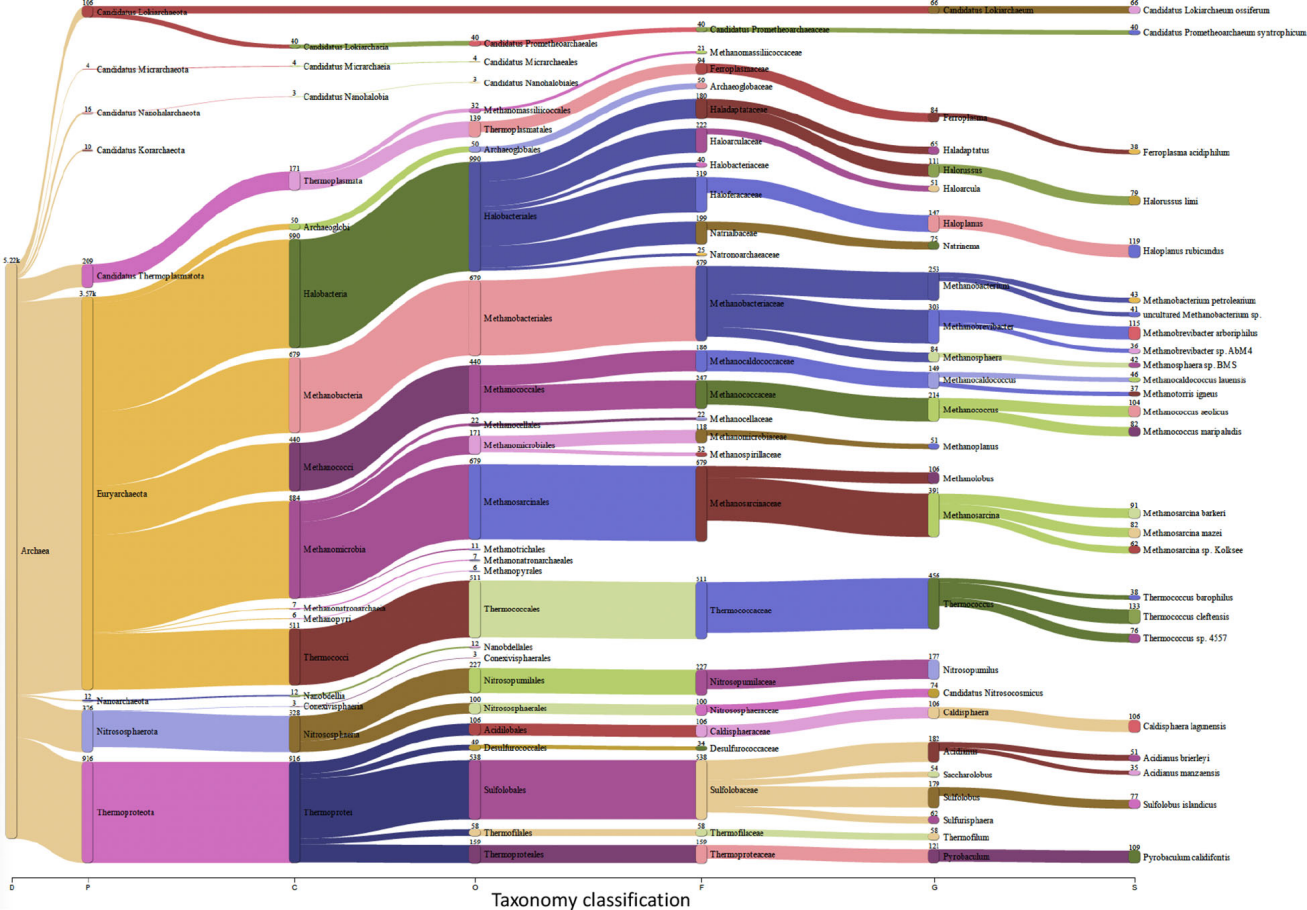
Archeal Communities in *Trialeurodes vaporariorum*. Sankey plot comprehensively illustrating, at various hierarchical taxonomic levels and after fitration at default threshold, the abundance and diversity of archeal communities harbored In *Trialeurodes vaporariorum*. D, Domain; P, Phylum; C, Class; O, Order; F, Family; G, Genus; S, Species.

**Figure 7:**
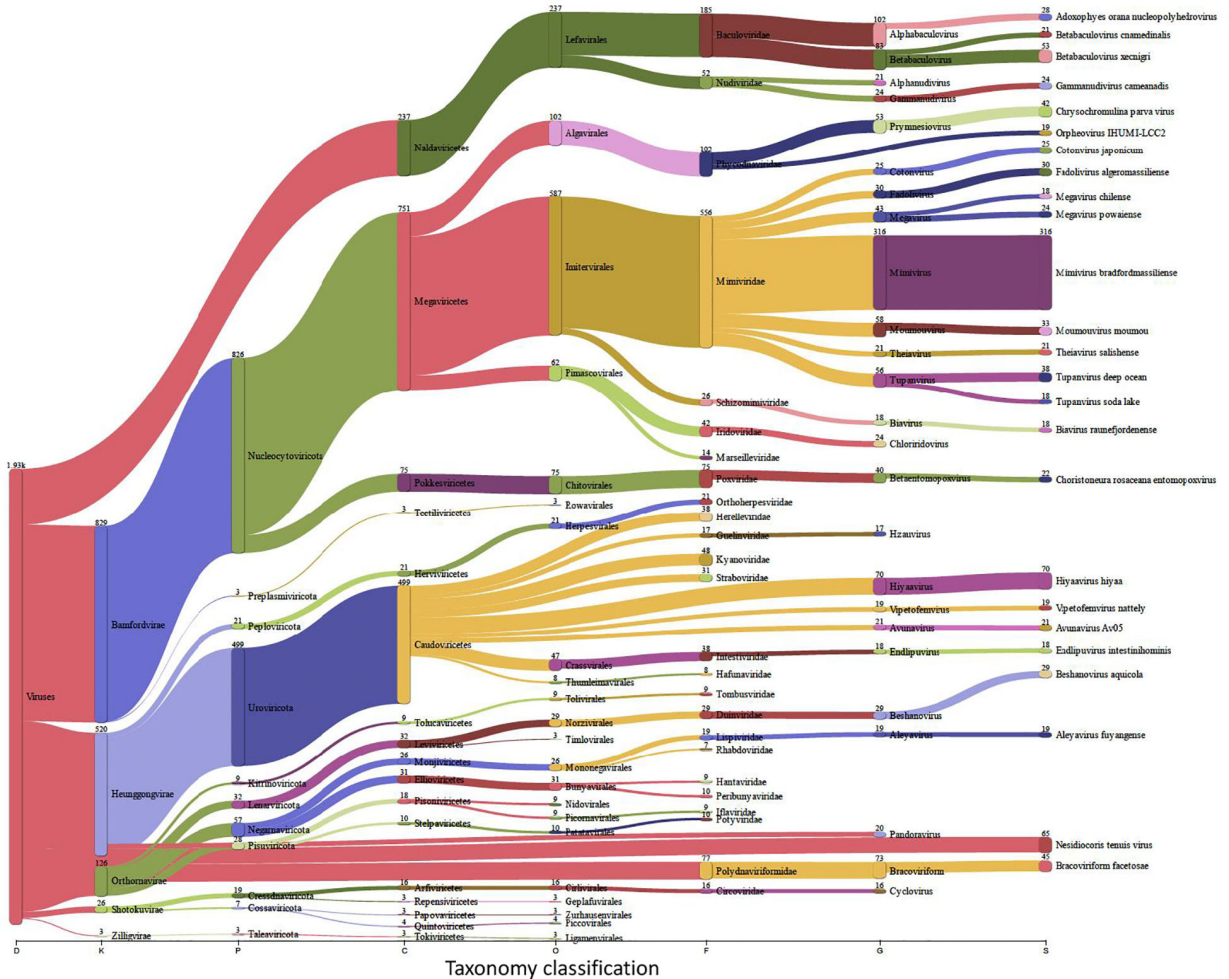
Viral Communities in *Traleurodes vaporariorum*. Sankey plot comprehensively illustrating, at various hierarchical taxonomic levels and after fitration at default threshold, the abundance and diversity of viral communities harbored in *Trialeurodes vaporariorum*. D, Domain; K, Kingdom; P, Phylum; C, Class; S, Species; O, Order; F, Family; G, Genus; S, Species.

**Figure 8:**
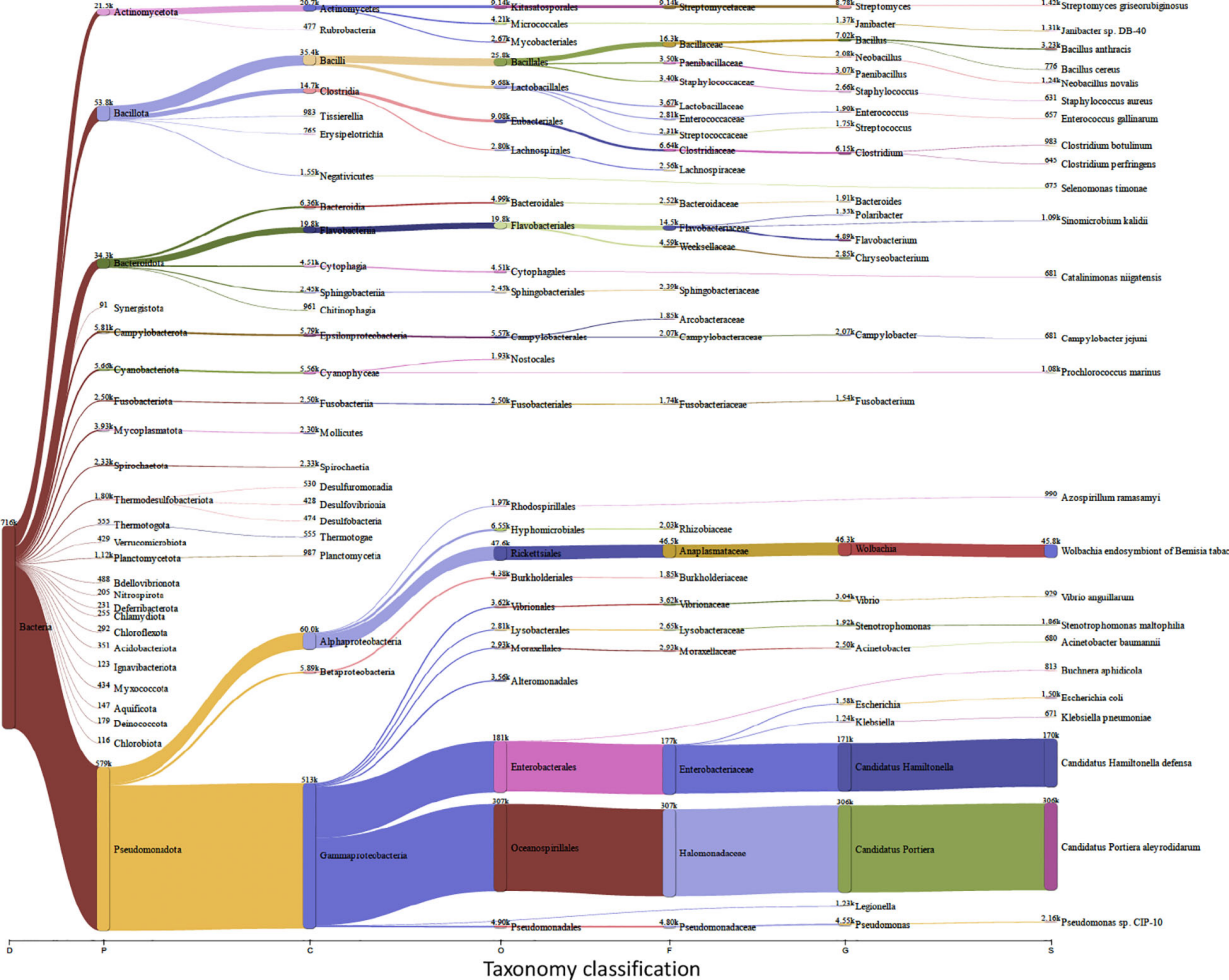
Bacterial Communities in *Trialeurodes vaporariorum*. Sankey plot comprehensively illustrating, at various hierarchical taxonomic levels and after fitration at default threshold, the abundance and diversity of harbored bacterial communities in *Trialeurodes vaporaniorum*. D, Domain; P, Phylum; C, Class; O, Order; F, Family; G, Genus; S, Species.

**Figure 9:**
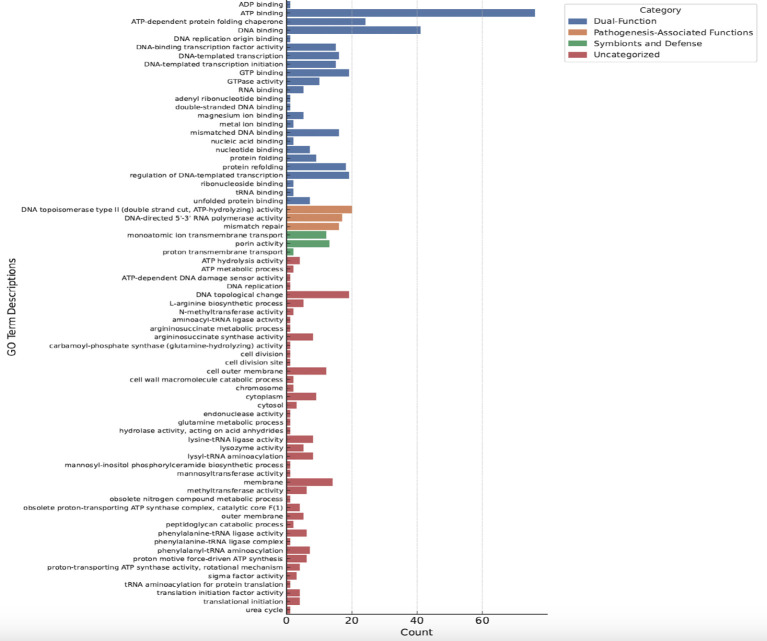
Gene ontology enrichment analysis of *Trialeurodes vaporariorum* metagenome. Histogram illustrates GO terms relevant to the genes in *Trialeurodes vaporariorum* metagenome.The y-axis represents the GO terms divided into four categories: blue (dual function),orange (pathogenesis-associated functions), green (symbionts and defense), and red (uncategorized). The x-axis represents the number of genes within each category. GO terms were considered enriched with a p-value cut-off of <0.05.

Insects, and like other living beings, generally harbor diverse groups of microbes including bacteria, viruses, fungi, archaea, and protozoa (Engel and Moran, 2013). These microbes could associate with the insect host either permanently or transiently and could be beneficial or harmful, therefore dictating the insect’s overall adaptability, survival aptitude and vector competence in various ecological niches (Chellappan and Ranjith, 2021). Along this notion, and to identify potential symbiotic or pathogenic microorganisms harbored in/carried by *TRIAVA* for self-protection against pathogens or for insect pathogenicity, we conducted a metagenomic analysis of *TRIAVA* and particularly scored for its Archaeal (**Figure 6**, **Supplementary Table 1**), viral (**Figure 7**, **Supplementary Table 2**), and bacterial (**Figure 8**, **Supplementary Table 3**) harbored communities. The calculated percentage of the abundance of archaeal, viral, and bacterial groups was determined by mapping the reads of each class to the total number of reads within each respective domain. For the archaeal community, the composition in *TRIAVA* was mainly dominated by members of the Euryarchaeota phylum, accounting for 3.57k reads (68.3%). The most abundant genera were *Thermococcus* (456 reads, 8.7%) and *Methanosarcina* (391 reads, 7.5%). Among these, the predominant species were *Thermococcus cleftensis* (133 reads), followed by *Thermococcus* sp. (76 reads), and *Thermococcus barophilus* (38 reads). Similarly, within *Methanosarcina*, the predominant species included *Methanosarcina bakeri* (91 reads), *Methanosarcina mazei* (82 reads), and *Methanosarcina* sp. *Kolksee* (62 reads). Likewise, *TRIAVA’s* viral community was also diverse, and mainly dominated by large dsDNA viruses, with Nucleocytoviricota (826 reads, 42.8%) emerging as the most abundant phylum. Within the Mimiviridae family, the genus *Mimivirus* (316 reads, 16.4%) was the most dominant. At the species level, *Mimivirus bradforsmassiliense* was the most prevalent (316 reads). Additionally, Urovicota (499 reads, 26%) was the second most abundant phylum. Within Caudoviricetes, the genus *Hiyaavirus* and the species *Hiyaavirus hiyaa* (70 reads, 3.6%) were the most abundant. Additionally, the bacterial community in *TRIAVA* was primarily composed of members of the Pseudomonadota phylum, which accounted for 579K reads (80.9%). The most dominant genera and species were *Candidatus Portiera aleyrodidarum* (306k reads, 42.7%), *Candidatus Hamiltonella defensa* (171k reads, 23.8%), followed by *Wolbachia endosymbionts* of *Bemisia tabaci* (46.3k reads, 6.3%). Furthermore, and to predict genes/proteins from the classified metagenome reads and interpret these gene sets, we also performed functional annotation and GO enrichment analysis (**Tables 2**, **3**; **Supplementary Table 4**; **Figure 9**). Our blastN findings revealed that the most prevalent species identified was *Candidatus Hamiltonella defensa*, with 81 genes aligned to the accession CP016303.1, 7 genes aligned to CP017613.1, 1 gene each for CP023988.1, CP017614.1, and CP017605.1. Additionally, *Candidatus Portiera aleyrodidarum* was also detected, CP016304.1 (7 genes). Various sequences matching *Bemisia tabaci* (another whitefly host) were also identified, including different RNA types: two non-coding RNAs (XR_002008634.1 and XR_002010031.1) and several mRNAs (XM_019058950.1, XM_019058068.1, XM_019052762.1, XM_019052620.1 and XM_019043942.1), each corresponding to one gene. Furthermore, we also identified complete and partial mitochondrial genome of *Bemisia tabaci* such as MH186145.1 (2 genes), XM_017766450.1 (1 gene), and MK360021.1 (1 gene). It is worth noting here that both *TRIAVA* and *Bemisia tabaci* are whiteflies and belong to the same family (Aleyrodidae), which means their genomes share significant sequence similarities. Since the genome of *Bemisia tabaci* is more extensively studied and well-annotated compared to *TRIAVA’s*, many of the sequences from *TRIAVA* could align to *Bemisia tabaci* simply due to evolutionary conservation rather than contamination. Additional sequences of interest included a match to *Plautia stali* symbiont (AP012551.1) with one gene. *Plautia stali* symbionts are typically associated with shield bugs, suggesting potential cross-contamination or shared microbial sequences. A sequence from *Cucumis melo* (LN683406.1) with one gene was also detected and this may indicate the presence of plant-originated sequences, potentially from environmental contamination. Lastly, *Wolbachia* endosymbiont of *TRIAVA* (CP016327.1) was also detected with one gene (**Table 2**). The percentage identity of the hits from the output for blastN (format 6) is provided in **Supplementary Table 5**.

**Table 2:** List of predicted genes from classified metagenomic reads.

#	Species	Accession	Number of Genes
1	*Candidatus Hamiltonella defensa* (*Bemisia tabaci*) strain MEAM1, complete genome	CP016303.1	81
2	*Candidatus Hamiltonella defensa* strain ZA17 chromosome	CP017613.1	7
3	*Candidatus Hamiltonella defensa* isolate MI12 chromosome 2, complete sequence	CP023988.1	1
4	*Candidatus Hamiltonella defensa* strain ZA17 plasmid pHDZA17.1	CP017614.1	1
5	*Candidatus Hamiltonella defensa* strain NY26 chromosome	CP017605.1	1
6	*Candidatus Portiera aleyrodidarum MED* (*Bemisia tabaci*) strain MEAM1, complete genome	CP016304.1	7
7	*Bemisia tabaci* uncharacterized LOC109029669 (LOC109029669), ncRNA	XR_002008634.1	1
8	*Bemisia tabaci* uncharacterized LOC109041975 (LOC109041975), transcript variant X2, ncRNA	XR_002010031.1	1
9	*Bemisia tabaci* probable peptidyl-tRNA hydrolase 2 (LOC109042289), transcript variant X4, mRNA	XM_019058950.1	1
10	*Bemisia tabaci* translation initiation factor IF-2-like (LOC109041659), mRNA	XM_019058068.1	1
11	*Bemisia tabaci* uncharacterized LOC109037904 (LOC109037904), partial mRNA	XM_019052762.1	1
12	*Bemisia tabaci* uncharacterized LOC109037803 (LOC109037803), mRNA	XM_019052620.1	1
13	*Bemisia tabaci* uncharacterized LOC109032038 (LOC109032038), transcript variant X2, mRNA	XM_019043942.1	1
14	*Bemisia tabaci* isolate 153_1 mitochondrion, complete genome	MH186145.1	2
15	*Bemisia tabaci* isolate Ortaca cytochrome oxidase subunit I (COI) gene, partial cds; mitochondrial	MK360021.1	1
16	*Plautia stali* symbiont DNA, complete genome	AP012551.1	1
17	*Cucumis melo* genomic scaffold, unassembled_sequence30944	LN683406.1	1
18	*Wolbachia* endosymbiont of *Trialeurodes vaporariorum*	CP016327.1	1

**Table 3:** Summarized list of identified protein domains in *Trialeurodes vaporariorum* microbiome linked to pathogenicity and symbiotic protection.

#	InterPro ID	Description	Count	GO Terms
1	IPR006626	Parallel beta-helix repeat	30	–
2	IPR011050	Pectin lyase fold/virulence factor	9	–
3	IPR000565	DNA topoisomerase, type IIA, subunit B	10	GO:0003677(InterPro)|GO:0003918(InterPro)|GO:0005524(InterPro)|GO:0006265(InterPro)
4	IPR004899	Pertactin, central region	5	–
5	IPR001525	C-5 cytosine methyltransferase	6	GO:0008168(InterPro)
6	IPR001387	Cro/C1-type helix-turn-helix domain	8	–
7	IPR001844	Chaperonin Cpn60/GroEL	18	GO:0042026(InterPro)|GO:0140662(InterPro)
8	IPR020575	Heat shock protein Hsp90, N-terminal	8	–
9	IPR013783	Immunoglobulin-like fold	17	–
10	IPR027417	P-loop containing nucleoside triphosphate hydrolase	13	–
11	IPR006315	Outer membrane autotransporter barrel domain	5	GO:0019867(InterPro)
12	IPR001702	Porin, Gram-negative type	6	GO:0009279(InterPro)|GO:0015288(InterPro)|GO:0016020(InterPro)|GO:0034220(InterPro)
13	IPR001897	Porin, gammaproteobacterial	6	GO:0009279(InterPro)|GO:0015288(InterPro)|GO:0016020(InterPro)|GO:0034220(InterPro)
14	IPR005483	Carbamoyl-phosphate synthase large subunit, CPSase domain	7	–
15	IPR005479	Carbamoyl-phosphate synthetase large subunit-like, ATP-binding domain	6	GO:0005524(InterPro)
16	IPR013656	PAS fold-4	1	–
17	IPR000792	Transcription regulator LuxR, C-terminal	3	GO:0006355(InterPro)

Along this, we identified a list of *TRIAVA* microbiome predicted protein domains encompassing a wide range of biological roles, including enzymes involved in various metabolic pathways, transporters facilitating the movement of molecules across cell membranes, DNA-binding domains participating in gene regulation, and chaperones assisting in protein folding and stability (**Supplementary Table 4**). Interestingly, several of those identified protein domains could be linked to pathogenicity and symbiotic protection (**Table 3**). The “Parallel beta-helix repeat” (IPR006626), a structural motif commonly found in enzymes such as pectate lyases that play a role in plant cell wall degradation, was among the most abundant domains detected in the *TRIAVA* microbiome. Furthermore, the “Chaperonin Cpn60/GroEL” (IPR001844), a molecular chaperone involved in protein folding and stress response, was also observed to be in high abundance. The “P-loop containing nucleoside triphosphate hydrolase” (IPR027417), an ATP-binding domain present in numerous enzymes, was also among the high abundant domains identified (**Table 3**). Along this, one of the most striking observations in the GO terms results (**Figure 9**) was the abundance of terms related to various DNA-binding and transcription-related processes. For instance, “DNA-binding transcription factor activity”, “regulation of DNA-templated transcription,” and “DNA-templated transcription initiation” were among the most highly represented terms. Another notable finding was also the presence of several GO terms associated with symbiosis and pathogenesis, such as “protein folding”, “ATP binding”, and “ADP binding”, highlighting further the intricate balance between beneficial and detrimental interactions within the *TRIAVA* microbiome. Remarkably as well, a significant number of GO terms that uniquely associate with symbiosis and defense mechanisms was detected. These include “lysozyme activity”, “lysyl-tRNA aminoacylation”, and “L-arginine biosynthetic process”. Also, several GO terms that could be linked to pathogenesis-associated functions exclusively like “phenylalanine-tRNA ligase activity,” “phenylalanine-tRNA ligase complex,” and “outer membrane” were also detected.

## Discussion

Greenhouse farming has been recently presented as the trail towards “precision agriculture” (Karanisa et al., 2022). With this bright prospect; however, and despite the positive impact of greenhouse farming on enhancing production and quality as compared to the traditional practice in open field systems, agronomists are still facing significant challenges (Maraveas et al., 2023; Farvardin et al., 2024; Yan et al., 2024). These challenges could be either due to natural driven factors like climate setpoint, wind, solar radiation, or static driven ones like defects in ventilation, screen, greenhouse homogenous structure (van Tuijl et al., 2008; Balendonck et al., 2010), and natural enemies including insect pests, plant pathogens, and weed (Oerke, 2006). Although environmental parameters are relatively maintained in greenhouse systems, fluctuations could still occur. In the cucumber and tomato grown sectors that we have studied, the recorded weekly average temperature and relative humidity readouts did fluctuate; yet, within standard ranges generally adopted for ideal production outcomes, with only a few, if any, readouts marginally either below or above these standard values (Peet et al., 1997; Miao, 2000; Kittas et al., 2005; Cherie, 2010; Boote et al., 2012; Hochmuth and Hochmuth, 2012; Jones, 2013; Shamshiri et al., 2018; Nikolaou et al., 2019; Amin et al., 2021). This; therefore, ruled out any potential negative impact of greenhouse environmental conditions on crop quality and yield in our studied sectors. Though ideal, establishing a “model climate” in greenhouses for production efficiency could; on the other hand, potentially impact the growth and abundance of insects in greenhouses, among which are pests that could cause crop damage and disease. In support of this outlook, the “insects survey” that we have conducted in the tomato and cucumber grown greenhouse sectors did indeed confirm the presence of various insect pests, including insect vectors, previously reported to cause crop diseases. One example of those pests is the whitefly, *TRIAVA*, known to transmit viruses like the *Tomato torrado virus* (Verbeek et al., 2014) and the *Pepino mosaic virus* (Noël et al., 2014) to tomato crops leading to severe necrosis and yield loss (van der Vlugt et al., 2000; Wieczorek et al., 2020). Another example is the *Aphis gossypii* aphid, a vector for more than fifty plant viruses including the *cucumber mosaic virus*, the *potato virus*, the *citrus tristeza virus*, and the *turnip virus* (Kennedy et al., 1962; Shim et al., 1979; Eastop VF, 2000), and a broadly dispersed pest for agricultural crops (Ebert and Cartwright, 1997; Im et al., 2022). Other insect vectors including *Creontiades pallidus* (Soyer, 1942; Wheeler, 2001; Zamharir and Shameli, 2023) and *Earias insulana* (Nasr and Azab, 1969; Borker et al., 1980) that transmit phytopathogenic pathogens to plants causing disease were also detected in our studied greenhouse sectors. Furthermore, insect pests reported to infest horticultural crops in open field and/or greenhouses systems in different parts of the world including *Liriomyza trifolii* (Spencer, 1973; Kang, 1996; Gao et al., 2017), *Hylastes cunicularius* (Zhou et al., 2004), *Bradysia* species (Hamlen and Mead, 1979; Jagdale et al., 2004; Vaughan et al., 2011), *Spoladea recurvalis* (Batra and Bhattacherjee, 1960; Aderolu, 2013; Mureithi et al., 2017; Othim et al., 2017; Othim et al., 2018), *Trichoplusia* species (Franklin et al., 2010; Cervantes et al., 2011), and *Diaphania indica* (Hutson, 1931; Soares, 1945; May, 1947) were also present in these studied cucumber and tomato grown sectors. It is worth noting here that insect collection in this study was carried out using only four tools, opening up for the potentiality of the presence of additional insects in these sectors that could have been probably missed.

To establish figurative alliance between insect presence and greenhouse environmental conditions, we correlated the abundance of *TRIAVA*, one of the identified insect pests/vectors in the studied greenhouse sectors, with greenhouse temperature and relative humidity conditions. Our findings revealed an overall positive correlation between *TRIAVA* abundance and temperature and a negative correlation between *TRIAVA* abundance and relative humidity. Comparable with our findings, previous studies conducted on whiteflies also delineated an inverse correlation between whiteflies population and precipitation and a direct correlation between whiteflies population and high temperature, with a fluctuation in the correlation trend between whiteflies population and humidity over several months of the year (Saghafipour et al., 2020). Other similar studies revealed no significant correlation between temperature and *TRIAVA* population and a negative correlation between rainfall rates and *TRIAVA* population (Nasruddin et al., 2021). In Gamarra et al’s adjusted temperature-dependent phenology predicted model, a temperature ranging from 11.5°C to 35.5°C was considered the typical range that allows *TRIAVA* development, with ~ 24°C being the ideal value within the range for a growth peak (Gamarra et al., 2020). It is important to highlight in this context that the impact severity of climate pressure on the insect is dependable on the adaptability and tolerance ability of the insect itself as well as on the amount of environmental stress exhibited. In some occurrences, climate conditions could also impact the microbial community residing within the insect, including the pathogens carried by the insect vectors, affecting the overall epidemic of a plant disease in an agricultural setting (Aregbesola et al., 2019). Here, we show that *TRIAVA* abundance, which adjacently concords with temperature and inversely correlates with relative humidity, negatively associates with crop yield, a finding that aligns with that of previous studies (Gamarra et al., 2016; Park et al., 1998; Bi et al., 2002; Moreau and Isman, 2012; Prijovic et al., 2013; Nasruddin and Mound, 2016; Kaya Apak et al., 2017).

Today, metagenomics has made significant advancements in agricultural practices. With the vast amount of generated data from metagenomics, the use of computational models based on environmental facets and changes in the microbial communities, has been allowing for more proactive pest control approaches. As such, and to better understand *TRIAVA’s* microbial community in such a scenery, conclude causal relationships between microbial presence and insect fitness, and particularly unravel whether the insect’s residing microbes could be potentially involved in *TRIAVA*’s self-protection mechanisms or in its pathogenic effect on crops, we comprehensively analyzed the metagenome of *TRIAVA* insects collected from the studied cucumber and tomato grown greenhouse sectors. Interestingly, the bacterial taxa detected in the *TRIAVA* metagenome highlighted the dominance of symbionts such as *Candidatus Portiera aleyrodidarum*, which is essential for nutrient provision to the host vector (Jiang et al., 2013), and other secondary symbionts such as *Candidatus Hamiltonella defensa* and *Wolbachia*. *Hamiltonella defensa* is known to harbor bacteriophages that encode toxins, which inhibit the larvae of parasitoid wasps (Degnan et al., 2009; Oliver et al., 2009). This symbiosis provides *TRIAVA* with enhanced survival capabilities in environments with high parasitoid pressure. *Wolbachia* modulates immune responses and may interfere with viral replication, this could; therefore, provide an indirect protection too (Hoffmann et al., 2011; Joubert et al., 2016). Previous studies have also shown that *Wolbachia* can reduce the ability of insect hosts to transmit certain viruses, which may be relevant to limiting the spread of begomoviruses (Kamtchum-Tatuene et al., 2017). Taken together, these symbionts detected in the *TRIAVA* microbiome support our postulate that the presence of symbiotic bacteria provides protective effects to the insect host.

Pathogenic bacterial species were also detected in *TRIAVA*’s studied microbiome. Examples of which include *Pseudomonas* spp., which are commonly associated with vascular wilt, blight and root diseases (AUSVEG; Durrands and Cooper, 1988; Hayward, 1991), *Stenotrophomonas* spp., known to be opportunistic pathogens (Gherardi et al., 2015; Lira et al., 2017), and *Flavobacterium* spp. which are often associated with tissue degradation in fish and could potentially cause rot (Rochat et al., 2019). *Streptomyces* spp., that could produce antibiotics (Ohnishi et al., 2008; Patzer and Braun, 2010) and cause scab-like symptoms through certain strains (Ismail et al., 2020; Ünlü and Elçi, 2022), were also detected in *TRIAVA*’s bacterial microbiome community. The presence of antibiotic-producing genera, such as *Streptomyces*, indicates the potentiality of microbial interference with external pathogenic bacteria. It is worth highlighting here that while *Streptomyces* spp. are more commonly associated with soil microbiomes (Gopalakrishnan et al., 2020), their low abundance in *TRIAVA* may still contribute to localized pathogen suppression. These symbionts likely form part of a broader defense mechanism that balances pathogen suppression and nutrient acquisition.

Relevant to *TRIAVA*’s identified viral community, the presence of the Geminiviridae family, albeit at low abundance, supports previous reports of *TRIAVA*’s aptitude to potentially serve as a vector for viral pathogens that cause significant economic losses. This family, for example, includes begomoviruses, such as *Tomato yellow leaf curl virus (TYLCV)*, which is a known pathogen for tomatoes (Wu et al., 2006; Stansly and Naranjo, 2010; Navas-Castillo et al., 2011; Yan et al., 2021). It is important to point out here that a key limitation in our metagenomic study is its exclusiveness in analyzing genomic DNA and not RNA samples which might have resulted in precluding the detection of RNA viruses present in *TRIAVA*’s viral community. Moreover, *TRIAVA*’s archaeal community was dominated by the phylum Euryarchaeota, which includes methanogenic and halophilic archaea. These archaea are typically found in saline and extreme environments (DasSarma et al., 2009). This is particularly relevant in Qatar, where the Persian Gulf has one of the highest salinity levels globally, reaching up to 50 PSU (practical salinity units) (Yao and Johns, 2010; Mohan et al., 2024). The detected halophilic archaea may originate from saline irrigation water used in greenhouses or from soil microbial communities affected by the environmental salinity. While archaea usually do not directly cause plant diseases (Cavicchioli et al., 2003), they may play a role in modulating the microbiome, affecting nutrient cycling and microbial interactions in the greenhouse ecosystem.

Along this, several of the identified protein domains linked to plant pathogenicity, insect virulence and invasion mechanisms, further support *TRIAVA*’s classification as a pest and a vector that can adversely affect plant health and crop yield. Among the identified domains, for example, the parallel beta-helix repeat (IPR006626) is commonly found in proteins such as pectin lyases known to degrade plant cell walls (Jenkins et al., 1998; Marín-Rodríguez et al., 2002) and is also reported to associate with bacteria that cause tissue degradation, such as *Pseudomonas* and *Erwinia* species (Weigele et al., 2003; Yamasaki et al., 2004; Abbott and Boraston Alisdair, 2008). Similarly, the pectin lyase fold/virulence factor (IPR011050) domain which indicates enzymatic activity to break down pectin, a major component of plant cell walls, potentially contributing to soft rot and vascular wilt diseases (Durrands and Cooper, 1988; Yoder et al., 1993; Mayans et al., 1997; de Sain and Rep, 2015), was also identified in our data set. The DNA topoisomerase, type IIA, subunit B (IPR000565) domain, which is essential for DNA replication and repair (Roca, 1995; Champoux, 2001; Wang, 2002; Corbett et al., 2005), was also identified. Its presence could potentially indicate bacterial replication mechanisms or prophage integration events, both of which can enhance virulence. Another significant domain is pertactin (IPR004899), which is commonly associated with bacterial adhesion and virulence (Charles et al., 1989; Li et al., 1992; Emsley et al., 1996). Pertactin-containing proteins help bacteria attach to host surfaces, facilitating colonization and infection (Locht, 1999). Additionally, the detection of the C-5 cytosine methyltransferase (IPR001525) domain suggests that certain bacteria in the metagenome may use DNA methylation to regulate gene expression or evade plant immune responses (Pósfai et al., 1988; Cheng et al., 1993; Goll et al., 2006; Ryazanova et al., 2012). Furthermore, the identification of the Cro/C1-type helix-turn-helix domain (IPR001387), typically found in regulatory proteins that control viral lytic and lysogenic cycles (Aggarwal et al., 1988; Steinmetzer et al., 2002), suggests the presence of prophages or bacteriophages that may contribute to horizontal gene transfer and enhance bacterial adaptability. Collectively, these findings highlight the potential presence of pathogenic bacteria and prophage elements that can contribute to plant damage. Our results also identified several protein domains that support the protective role of symbiotic bacteria in the whitefly microbiome. The chaperonin Cpn60/GroEL (IPR001844) domain was among the most common. Chaperonins are molecular chaperones that assist in protein folding and are crucial for maintaining protein stability under stressful conditions (Hemmingsen et al., 1988; Hill et al., 2004; Chaurasia and Apte, 2009). Symbionts like *Hamiltonella defensa* and *Wolbachia* may use these chaperonins to enhance their resilience and support the whitefly host during environmental stress. Another identified key protein domain is the heat shock protein Hsp90, N-terminal (IPR020575) domain, which plays a role in stabilizing regulatory proteins during stress responses (Zuehlke and Johnson, 2010; Johnson, 2012; Lavery et al., 2014). Its identification could suggest that symbionts or *TRIAVA* itself may rely on heat shock proteins to survive in certain environmental conditions, such as the greenhouse temperature conditions. The immunoglobulin-like fold (IPR013783), structurally similar to immune-related proteins (Bork et al., 1994; Halaby et al., 1999; Potapov et al., 2004), was also detected, indicating the presence of proteins that may participate in defense-like responses, potentially modulating interactions with pathogens. The P-loop containing nucleoside triphosphate hydrolase (IPR027417) domain, commonly associated with ATPases, was also pronounced in our results. ATPases play crucial roles in energy-dependent processes such as signal transduction and defense mechanisms (Walker et al., 1982; Leipe et al., 2002; Leipe et al., 2004). Symbionts may; therefore, use these ATP-dependent proteins to maintain their protective functions. Additionally, the presence of the outer membrane autotransporter barrel domain (IPR006315) suggests that symbionts may secrete effector proteins to modulate the host’s immune response or inhibit pathogenic bacteria (Henderson et al., 1998; Lee and Schneewind, 2001; Meuskens et al., 2019). Two porin-related domains, Gram-negative type porins (IPR001702) and gammaproteobacterial porins (IPR001897), were also detected in our results. Porins mediate the transport of molecules across bacterial membranes, which can regulate nutrient uptake and the release of molecules involved in microbial competition (Jap and Walian, 1990; Pauptit et al., 1991; Kreusch and Schulz, 1994; Galdiero et al., 2012). These porins may thus enable symbionts to control their internal environment or secrete factors that suppress pathogens. We also identified the carbamoyl-phosphate synthase/synthetase domains (IPR005483, IPR005479), involved in nitrogen metabolism (Stapleton et al., 1996; Raushel et al., 1999; Kim et al., 2002; Saha et al., 2007; Schoneveld et al., 2007), suggesting that symbionts may contribute to nitrogen cycling, potentially enhancing whitefly survival by supplementing essential nutrients. Another interesting protein domain detected in our findings is the Signal Transduction and Environmental Sensing, PAS Fold-4 (IPR013656). PAS domains are known for their roles in sensing environmental stimuli such as oxygen, redox potential, and light (Ponting and Aravind, 1997; Zhulin et al., 1997; Hefti et al., 2004). Therefore, the presence of these domains suggests that symbionts (like *Hamiltonella* and *Portiera*) may help the vector detect environmental stressors, such as reactive oxygen species (ROS), known to be part of a plant’s immune defense mechanisms. Proteins with PAS domains could regulate such stress responses, helping the symbionts and the whitefly vector adapt to harsh environments that could occur in greenhouses. Additionally, protein domains relevant to the Transcription regulator LuxR, C-terminal (IPR000792), involved in quorum sensing, were also detected (Ducros et al., 2001; Maris et al., 2002). Quorum sensing may allow symbionts like *Hamiltonella* to coordinate toxin production when parasitoids or other threats are present. LuxR proteins could also regulate genes involved in nutrient exchange between the symbionts and the whitefly. Taken together, the presence of transcriptional regulators (like LuxR family) and environmental sensors (like PAS domains) indicates that symbionts may modulate the host’s immune system, reducing susceptibility to pathogens. This could also explain how symbionts like *Wolbachia* interfere with viral replication (Chrostek et al., 2013; Rainey et al., 2016). The detection of PAS and LuxR domains could also support the postulate that symbionts regulate their defense mechanisms and potentially enhance *TRIAVA*’s resilience by sensing environmental stressors, coordinating protective responses through quorum sensing and modulating stress and immune responses to reduce ROS-related damage. Collectively, these computational predictions set the stage for further experimental validations to elucidate insects-microbial interactions in future studies.

## Conclusion

In agro-ecological systems, the interaction between climate conditions, multi-species and the grown crops is complex and multifaceted, complicating in many occurrences the development of robust, smart, and controlled agricultural production strategies. In this study, we provide further insight into the rapports between meteorological circumstances, insects, and crop yield in greenhouses, the findings of which will serve ongoing efforts to sail towards an upsurge in agricultural production and surpass food insecurity.

## Data Availability

The metagenome assembly data for this study have been deposited in NCBI BioProject under accession number PRJNA1216413.
